# Fumanjian, a Classic Chinese Herbal Formula, Can Ameliorate the Impairment of Spatial Learning and Memory through Apoptotic Signaling Pathway in the Hippocampus of Rats with **A*β*_1–40_**-Induced Alzheimer's Disease

**DOI:** 10.1155/2014/942917

**Published:** 2014-06-22

**Authors:** Hai-yan Hu, Zhi-hui Cui, Hui-qin Li, Yi-ru Wang, Xiang Chen, Ji-huang Li, Dong-mei Xv, Guo-qing Zheng

**Affiliations:** ^1^The Second Clinical Medical College of Wenzhou Medical University, Wenzhou 325027, China; ^2^Center of Neurology and Rehabilitation, The Second Affiliated Hospital & Yuying Children's Hospital of Wenzhou Medical University, Wenzhou 325027, China

## Abstract

Alzheimer's disease (AD) is the most common form of dementia and lacks disease-altering treatments. Fumanjian (FMJ), a famous classic Chinese herbal prescription for dementia, was first recorded in the *Complete Works of Jingyue* during the Ming Dynasty. This study aimed to investigate whether FMJ could prevent cognitive deficit and take neuroprotective effects in A*β*_1–40_-induced rat model through apoptotic signaling pathway. AD model was established by bilateral injection of A*β*_1–40_ into hippocampus in rat. All rats were tested for their capabilities of spatial navigation and memorization by Morris water maze. Apoptosis was tested using TUNEL staining in hippocampus neuronal cells; RT-PCR tested expression of Bcl-2 and Bax mRNA; western blotting tested protein level of cleaved caspase-3. After 14 days of treatment, FMJ significantly improved the escape latency and enhanced platform-cross number compared with the A*β*_1–40_-injected group (*P* < 0.05 or *P* < 0.01). FMJ also significantly decreased number of TUNEL-positive neuronal apoptosis and the expressions of Bax and cleaved Caspase-3 and increased the expression of Bcl-2 (*P* < 0.01) compared with AD model group. In conclusion, FMJ exerts a protective effect against A*β*_1–40_-induced learning and memory deficits and neuronal apoptosis, suggesting that FMJ could be used as a potential therapeutic formula for AD.

## 1. Introduction

Alzheimer's disease (AD) is a devastating and age-related neurodegenerative disorder and is the most common cause of dementia that affects more than 37 million people worldwide [1]. The economic burden of AD is monstrous;* the World Alzheimer Report 2013* [2] estimated that the worldwide cost of dementia care is currently over US$600 billion or around 1% of global GDP. Even worse, at present there are only symptomatic therapies in nature, providing modest improvements in memory without altering the progression of the disease pathology [1, 3]. In China, the prevalence of Alzheimer's and dementia increased substantially between 1990 and 2010, and the burden of dementia might have been underestimated by double [4]. Fortunately, the most appreciable distinction between China and the West in treating PD is the use of traditional Chinese medicine (TCM) therapy, which includes Chinese herbal medicine (CHM), acupuncture, and other nonmedication therapies. The important characteristic of China's national medical system is that TCM and Western medicine complement and cooperate with each other, being responsible together for the health care of Chinese people [5]. Given the high incidence of AD and the relative poverty of conventional treatment, many researchers thus resort to CHM and herbal compound as potential agents to prevent the progression of AD [6, 7].

TCM has been practiced for a 3000 years' history of human use. Jingyue Zhang (1563–1640; original name: Jiebin Zhang) had an immense influence on the development of Chinese medicine during the Ming Dynasty. Zhang's famous work was Jingyue Quanshu (*Complete Works of Jingyue*), which summed up the therapeutic concepts and formulations not only of himself, but also of his predecessors. In his great achievement book, Jingyue Zhang recorded 21 formulae for improving cognitive function [8]. Fumanjian (FMJ), a famous classic Chinese herbal prescription for dementia, was first recorded in the Jingyue Quanshu [9]. In modern times, FMJ is still used continuously and widely for treatment of dementia [10–12]. However, the therapeutic mechanism of FMJ against dementia still remains unclear.

Aggregation of amyloid-*β* (A*β*) peptides is the crucial factor in the onset of AD. The deposition of aggregated A*β* triggers a cellular stress response called the unfolded protein response (UPR); the UPR signaling pathway is a cellular defense system for dealing with the accumulation of misfolded proteins but switches to apoptosis when endoplasmic reticulum stress is prolonged [13]. Neuronal apoptosis, a genetically programmed form of neuronal death, plays an important role in AD [14]. Therefore, this study aims to investigate whether FMJ could prevent cognitive deficit and take neuroprotective effects in A*β*_1–40_-induced rat model of AD through apoptotic signaling pathway.

## 2. Materials and Methods

### 2.1. Experimental Animals

Fifty six Sprague-Dawley (SD) rats of SPF grade, weighing 230–270 g, were provided by the Beijing Vital River Laboratory Animal Technology Co., Ltd., China (Certification No: SCXK 2012-0001, Beijing), and bred in the Laboratory Animal Center of Wenzhou Medical University. Animals were housed in a room with temperature of 23–25°C, relative humidity of 50–60%, and a 12 h light/12 h dark cycle (lights on at 08:00 h). They had free access to common forage and water. All animal experiments were conducted in accordance with the Guide for the Care and Use of Laboratory Animals issued by National Academy of Sciences, Institute of Laboratory Animal Resources, Commission on Life Sciences, and National Research Council. All procedures used in this study were approved by the local ethical committee for animal research.

### 2.2. Drugs and Reagents

FMJ is composed of ten kinds of CHMs: (A) Radix Rehmanniae Recens; unprocessed rehmannia root (*shengdihuang*), the dried roots of Radix Rehmanniae Recens; (B) Radix Ophiopogoni; dwarf lilyturf tuber (*maidong*), the dried roots of Ophiopogon japonicus (Thunb.) Ker-Gawl.; (C) Radix Paeoniae Alba; debark peony root (*baishao*), the dried roots of* Paeonia lactiflora* Pall.; (D) Rhizoma acori tatarinowii; grassleaf sweetflag rhizome (*shichangpu*), the dried roots of Acorus tatarinowii Schott; (E) Herba Dendrobii; Dendrobium (*shihu*), the dried roots of* Dendrobium officinale* Kimra et Migo.; (F) Cortex Moutan Radicis; tree peony root bark (*mudanpi*), the dried root barks of* Paeonia suffruticosa* Andr.; (G) Poria; Indian bread (*fuling*), the dried Sclerotia of Poria cocos (Schw.) Wolf; (H) Pericarpium Citri Reticulatae; dried tangerine peel (*chenpi*), the dried fruit peels of Citrus reticulata Blanco; (I) Caulis Akebiae; akebia stem (*mutong*), the dried stems of Akebia quinata (Thunb.) Decne.; and (J) Rhizoma Anemarrhenae; common anemarrhena rhizome (*zhimu*), the dried roots of* Anemarrhena asphodeloides* Bge., in the ratio of 2 : 2 : 2 : 2 : 2 : 2 : 2 : 1 : 1.5 : 1.5 on a dry weight basis, respectively, all of which are recorded in the Chinese Pharmacopoeia. We used FMJ in the present study minus Caulis Akebiae and replaced Radix Sophorae Flavescentis (*kucan*), the dried roots of* Sophora flavescens* Ait, because Caulis Akebiae may cause potentially renal damage. All herb ingredients were provided by the Second Affiliated Hospital of Wenzhou Medical University and verified by the Department of Chinese Materia Medica of Wenzhou Medical University. After decocted with appropriate amounts of water, extracted twice, filtered, and concentrated, the raw herbs were made into 1 g/mL stock solution. The stock solution was stored at 4°C until use. Donepezil (Aricept) was purchased from the Eisai Pharmaceutical Co., Ltd. (Batch number 100223A) and used as positive control.

### 2.3. A*β*_1–40_-Induced AD Model and Experimental Design

A*β*_1–40_ was incubated according to the manufacturer's instructions to allow the change in an assembly state of the peptide with ensuing toxicity. After anesthetized by intraperitoneal injection of 10% chloral hydrate at a dosage of 3-4 mL/kg, the rats were mounted in a stereotactic frame and injected by pressure with the incubated A*β*_1–40_ solution into each side of the hippocampus with the volume of 2 *μ*L containing 5 *μ*g A*β*_1–40_ using the following stereotaxic coordinates [15]: 3.0 mm posterior to the bregma, 1.5 mm left/right to the midline, and 2.8 mm ventral to the bregma. The injection was performed within 5 min after the injection; the needle remained in the target location for 10 min to avoid the tracer reflux along the needle tract. Sham-operation rats took the same operation as A*β*_1–40_-injected animals except that injection of the same volume of saline instead of A*β*_1–40_.

In the present study, we selected 4.75 mg/kg/day, 9.5 mg/kg/day, and 19 mg/kg/day of FMJ as the low, medium, and high dosages, respectively. Rats were randomly divided into 7 groups with 8 rats per group: normal control, sham-operation group, A*β*_1–40_-injected group, A*β*_1–40_ plus Aricept group, A*β*_1–40_ plus FMJ 4.75 mg/kg, A*β*_1–40_ plus FMJ 9.5 mg/kg, and A*β*_1–40_ plus FMJ 19 mg/kg. The rats in the FMJ-treated groups were orally administered with the corresponding doses of FMJ, A*β*_1–40_ plus Aricept group were orally administered with Aricept at 1.67 mg/kg/day, and normal control group, sham-operation group, and A*β*_1–40_-injected group were given the same volume of normal saline.

### 2.4. The Morris Water Maze Detection

Spatial learning and memory of rats was assessed in Morris water maze as published previously [16] by two investigators completely blind to the treatment of the animals. The Morris water maze consisted of a circular pool (160 cm diameter and 50 cm deep) filled with warm water (23 ± 1°C) to a depth of 27 cm and an escape platform (10 cm diameter) submerged 1 cm below the surface of the water. Swimming activity of the rats was monitored via a video camera mounted overhead and automatically recorded via a video tracking system.

At the beginning of each trial, the rats were placed into the water facing the wall of the pool at one of the four quadrants. Each rat was allowed 60 s to find and mount the platform. When the rat found the platform, it was kept on the platform for 10 s. The rats were given four trials per day and the average time to find the platform of the 4 trials (escape latency) was recorded by video tracking software. The rats were then towel dried and placed in a cage with a heating pad underneath until dry and returned to their home cage. The training session was conducted for 4 consecutive days in which the platform was never moved. The shorter escape latency, the stronger spatial learning ability.

Memory retention was evaluated by the platform-cross number in a probe trial. On the 5th day, the platform was removed. In this probe trial, the rats were put into the pool and allowed to swim freely in the pool for 60 s. The number of crossings of the location of the platform was recorded.

Working memory was evaluated by the Trail1/Trail4 index. On the 6th, 7th, and 8th days, we placed the platform on the opposite quadrants. The rats were put into the pool and the total distance traveled was recorded until the rats found the platform. The rats were given 4 consecutive trials every day, and the interval between 2 trials was less than 2 min. The distance was recorded as trail 1, trail 2, trail 3, and trail 4, and the ratio from trail 1 to trail 4 (Trail1/Trail4) indicates the ability of working memory.

### 2.5. Apoptosis Assessment

To detect cells undergoing apoptosis, TUNEL technique was performed according to the manufacturer's protocol supplied within the TUNEL-pod kit (Roche, USA). At first, the brain sections of hippocampus CA1 region were immersed in 3% H_2_O_2_ for inactivation of endogenous hydrogen peroxidase activity. After rinsing with PBS, the sections were incubated with proteinase K solution at 37°C for 20 min to enhance the permeability. Then, the sections were incubated for 60 min with TUNEL reaction mixture and for 30 min with converter-POD at 37°C. At last, after incubated for 10 min with DAB substrate solution (Zymed, USA), the sections were counterstained with 0.5% methyl green. Positive and negative controls were carried out on slides from the same block. Stained slides were randomly observed under a high magnification microscope and the pathological changes near the injection needle were photographed. The image pictures were processed by the America IPP6.0 software and the positive cells (brown) were calculated.

### 2.6. RT-PCR

Quantitative gene expression was measured by real-time PCR. Total RNA from the hippocampus was isolated with TriZol reagent (Shanghai ShengGong biological engineering co., LTD) and cDNA was synthesized with reverse transcriptase (The Bioneer). All the PCR primers were designed and configured by the Shanghai Rui Jingsheng Biological Engineering Co., Ltd. The primers sequences for Bcl-2 were 5′ GTGAACTGGGGGAGGATTGT 3′ (sense) and 5′ GCATCCCAGCCTCCGTTA 3′ (antisense). The primer sequences for Bax were 5′ CCCGAGAGGTCTTCTTCCG 3′ (sense) and 5′ GAAGTCCAGTGTCCAGCCCA 3′ (antisense). The primer sequences for *β*-actin were 5′ CCCATCTATGAGGGTTACGC 3′ (sense) and 5′ TTTAATGTCACGCACGATTTC 3′ (antisense). The real-time PCR conditions were as follows: initial denaturation at 94°C for 4 min followed by 94°C for 20 s, 60°C for 30 s, and 35 cycles of 72°C for 30 s. The data were analyzed by Software 2.2 using Ct value as the readout and normalized relative to levels of *β*-actin.

### 2.7. Western Blot Analysis

Expression of cleaved caspase-3 was analyzed by western blot and quantified by the BCA protein assay kit (Pierce, USA). The frozen hippocampus samples were ground and lysed in RIPA lysis buffer containing a protease inhibitor cocktail (Roche). Forty micrograms protein of each sample was separated by 10% SDS-PAGE gels and then transferred onto polyvinylidene difluoride (PVDF) membrane. Blotting membranes were blocked for 1 h at room temperature and probed with corresponding primary antibodies overnight at 40°C. After incubation with horseradish peroxidase-conjugated goat anti-rabbit secondary antibody for 2 h at room temperature, bands were visualized using a chemiluminescence-based detection kit (Pierce Company, USA) and the OD value was quantified using Quantity One gel analysis system quantitated by densitometry.

### 2.8. Statistical Analysis

All values were expressed as mean ± standard deviation ($\overset{-}{x}\pm \text{s}$). Normality test was performed to all data (*P* > 0.1 prompts normal distribution). The escape latency and Trail1/Trail4 at the same time points were analyzed by repeated measures analysis of variance (RANOVA). All other assessments were analyzed using the signal factor analysis of variance (one-way ANOVA). Pair wise comparison was performed using LSD test for homogeneous variance, and Dunnett's T3 test for nonhomogeneous variance. In all cases, *P* < 0.05 was considered significant. All data were processed using SPSS16.0 statistics software.

## 3. Results

### 3.1. Fumanjian Prevented A*β*_1–40_-Induced Impairment of Spatial Learning and Memory

**Figure 1:**
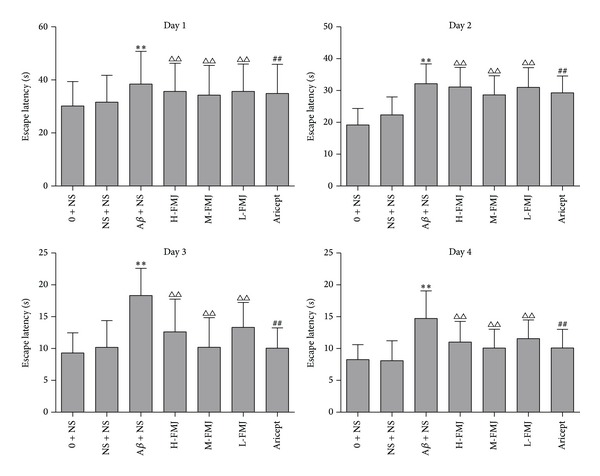
The comparison of escape latency in rats. **P < 0.01, compared with sham-operation group in the same time point; ^△△^ *P* < 0.01, compared with A*β* + NS group in the same time point; ^##^ *P* < 0.01, compared with A*β* + NS group in the same time point. NS: normal saline; A*β*: amyloid-*β*; H-FMJ: Fumanjian high dosage group; M-FMJ: Fumanjian medium dosage group; L-FMJ: Fumanjian low dosage group.

**Figure 2:**
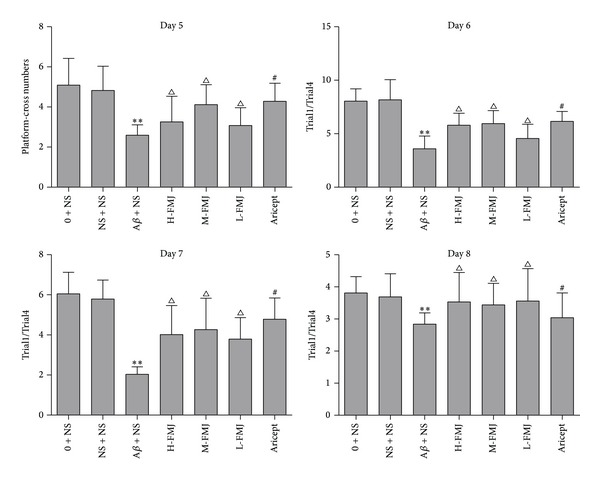
The comparison of platform-cross numbers and Trail1/Trail4 in rats. **P < 0.01, compared with sham-operation group in the same time point; ^△^ *P* < 0.05, compared with A*β* + NS group in the same time point; ^#^ *P* < 0.05, compared with A*β* + NS group in the same time point. NS: normal saline; A*β*: amyloid-*β*; H-FMJ: Fumanjian high dosage group; M-FMJ: Fumanjian medium dosage group; L-FMJ: Fumanjian low dosage group.

In the Morris water maze test, all animals were able to swim normally and find the hidden platform during the training trials. After being trained four times per day for four consecutive sessions, normal control and sham-operation rats were able to reach the hidden platform in a shorter time during the training. However, the learning and memory abilities of A*β*_1–40_-injected rats were significantly impaired compared with the sham-operation group (*P* < 0.01) (Figure 1). A significant decrease in escape latency was observed in the Aricept or FMJ group compared with the A*β*_1–40_-injected group (*P* < 0.01) and at dose of 9.5 mg/kg/d showed the maximum decline (Figure 1). In the probe trial of the Morris water maze test, injection of A*β*_1–40_ had a significant effect on the platform-cross number compared with the sham-operation group (*P* < 0.01). Compared with the A*β*_1–40_-injected group, Aricept or FMJ-treated rats displayed more platform-cross number (*P* < 0.05) Figure 2. In the Morris water maze test, injection of A*β*_1–40_ had a significantly lower effect on the Trail1/Trail4 index compared with the sham-operation group (*P* < 0.01). Compared with the A*β*_1–40_-injected group, Aricept or FMJ-treated rats displayed higher Trail1/Trail4 index (*P* < 0.05) Figure 2.

### 3.2. Fumanjian Suppressed A*β*_1–40_-Induced Apoptosis in the Hippocampus CA1 Area

**Figure 3:**
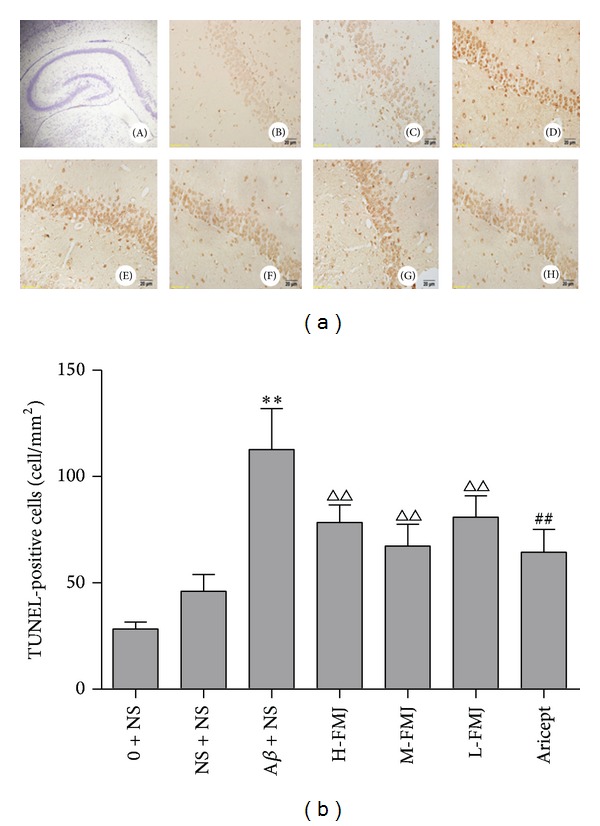
Representative photomicrographs of TUNEL staining in the hippocampus CA1 area of each group (×400). (A) 100 × hippocampus; (B) normal control group; (C) sham-operated group; (D) model group; (E) Fumanjian high dosage group; (F) Fumanjian mid-dosage group; (G) Fumanjian low dosage group; (H) Aricept positive control group. Quantitative analysis showed that treatment group reduced the number of TUNEL-positive cells in the hippocampus CA1 area. **P < 0.01, compared with sham-operation group; ^△△^ *P* < 0.01, compared with NS + A*β* group; ^##^ *P* < 0.01, compared with NS + A*β* group. NS: normal saline; A*β*: amyloid-*β*; H-FMJ: Fumanjian high dosage group; M-FMJ: Fumanjian medium dosage group; L-FMJ: Fumanjian low dosage group.

Sections through the hippocampus CA1 area were examined via TUNEL assay. Microscopic inspection of the hippocampal sections from normal control and sham-operation rats revealed morphologically normal neurons with no TUNEL reaction. After the injection of A*β*_1–40_, a significant number of TUNEL-positive pyramidal neurons were detected in the CA1 subfield of the hippocampus compared with sham-operation rats (*P* < 0.01). Aricept or FMJ significantly reduced the number of TUNEL-positive neurons compared with A*β*_1–40_-injected group (*P* < 0.01) Figure 3.

### 3.3. Fumanjian Reversed A*β*_1–40_-Induced Alteration of Expression of Bcl-2 and Bax mRNA in the Hippocampus CA1 Area

**Figure 4:**
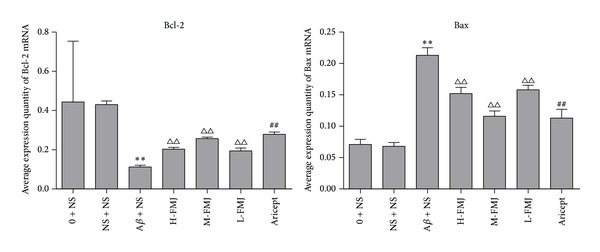
Comparison of Bcl-2 and Bax mRNA levels in the hippocampus. **P < 0.01, compared with sham-operation group; ^△△^ *P* < 0.01, compared with A*β* + NS group; ^##^ *P* < 0.01, compared with A*β* + NS group. NS: normal saline; A*β*: amyloid-*β*; H-FMJ: Fumanjian high dosage group; M-FMJ: Fumanjian medium dosage group; L-FMJ: Fumanjian low dosage group.

The effects of FMJ on the expression of Bcl-2 and Bax mRNA were investigated by real-time RT-PCR. Compared with the sham-operation group, injection of A*β*_1–40_ in the hippocampus highly increased the expression of Bax mRNA and decreased the expression of Bcl-2 mRNA (*P* < 0.01) Figure 4. Compared with the injection of A*β*_1–40_ group, Aricept or FMJ significantly decreased the expression of Bax mRNA and increased the expression of Bcl-2 mRNA (*P* < 0.01) Figure 4.

### 3.4. Fumanjian Lowered Protein Levels of Cleaved Caspase-3 in the Hippocampus CA1 Area

**Figure 5:**
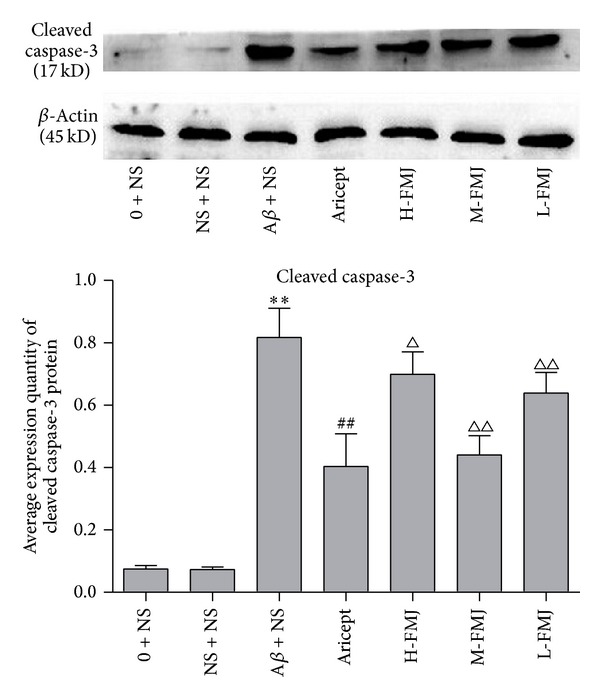
The protein levels of cleaved caspase-3 in the hippocampus CA1 area. **P < 0.001, compared with sham-operation group; ^△^ *P* < 0.05 and ^△△^ *P* < 0.01, compared with A*β* + NS group; ^##^ *P* < 0.01, compared with A*β* + NS group. NS: normal saline; A*β*: amyloid-*β*; H-FMJ: Fumanjian high dosage group; M-FMJ: Fumanjian medium dosage group; L-FMJ: Fumanjian low dosage group.

The protein levels of cleaved caspase-3 in the hippocampus CA1 area were investigated using western blot. Compared with the sham-operation group, the protein levels of cleaved caspase-3 were significantly increased after injection of A*β*_1–40_ (*P* < 0.01) Figure 5. After treatment with Aricept or FMJ, the protein levels of cleaved caspase-3 were significantly decreased at different degrees (*P* < 0.05 or *P* < 0.01) Figure 5. Among the three doses of Manjian, the decline of Caspase-3 in Manjian 9.5 mg/kg rats showed the maximum, which was comparable to Aricept positive control.

## 4. Discussion

In the present study, we found that FMJ can ameliorate the spatial memory impairment through apoptotic signaling pathway, which could increase the expression of Bcl-2 and downregulate the expression of Bax and caspase-3 in the hippocampus of rats with A*β*_1–40_-induced Alzheimer's disease.

Proteolytic processing enzymatically of transmembrane amyloid precursor protein (APP) forms A*β* peptides, a 39~43 amino acid peptides [17]. According to amyloid hypothesis, the deposition of aggregated A*β* in brain areas involved in cognitive functions is assumed to initiate a pathological cascade resulting in neuronal dysfunction and death in AD [18, 19]. Numerous reports have indicated that the injection of A*β*_1–40_ into rat hippocampus provides an effective model to impair memory and to elicit the pathologic changes of AD [20–22] because A*β*_1–40_ aggregates play an important role in the pathogenesis of AD [23]. In the present study, we demonstrated that A*β*_1–40_ injection can induce the impairment of spatial learning and memory and FMJ treatment can also effectively ameliorate A*β*-induced memory deficits.

The deposition of A*β* peptide can induce neuronal apoptosis, which is an essential cellular process and a major pathway for neuronal death in neurodegeneration in AD [24]. Apoptotic signaling is generally classified as proceeding by either an intrinsic pathway or an extrinsic pathway. The intrinsic apoptotic signaling pathway is a crucial pathway in neuronal loss in AD. The interaction between the antiapoptotic protein Bcl-2 and the proapoptotic protein Bax plays a key role in the activation of intrinsic apoptotic signals involving mitochondria which secondary release cytochrome C and the activation of intracellular cysteine proteases, particularly caspase-9 and caspase-3. The initiator caspase-9 is activated and can initiate the activation of executioner caspase, mainly caspase-3, resulting in cell destruction by proteolysis [25]. In the present study, we presented evidences that the neuronal damage induced by infusing A*β*_1–40_ into hippocampus proceeded through apoptotic signaling pathway and FMJ attenuated apoptotic-like changes.

Caspases have a direct role in APP processing and in the biogenesis of A*β* peptide species [26]. Caspase-3 activation has been identified as a crucial event of neuronal programmed cell death program in AD [27]. The Bcl-2 protein family consists of proapoptotic and antiapoptotic members that interact at both the physical and the functional levels to regulate mitochondrial integrity and apoptotic cell death [28]. Bcl-2 and Bax are regulated by the mitochondrial pathway, which are considered to be an important step in controlling and initiating apoptosis in AD [29]. In the present investigation, we demonstrated that FMJ enhanced the expression of the antiapoptotic protein Bcl-2 and decreased the expression of the proapoptotic protein Bax, thus resulting in a reduction of caspase activation.

In FMJ for dementia, four TCM herb ingredients including Rhizoma acori tatarinowii, Radix Rehmanniae, Poria cocos, and Radix Ophiopogonis were listed top 10 TCM herb ingredients for highest potential benefit to dementia intervention according to a literature survey that related to the highest frequency of use in 236 formulae collected from 29 ancient Pharmacopoeias, ancient formula books, or historical archives on ancient renowned TCM doctors, over the past 10 centuries [8]. The pharmacological mechanisms of individual herb ingredients or responsible active principles of FMJ for the cognitive benefits are summarized as follows. (1) Catalpol, an iridoid glycoside, was isolated from the fresh Radix Rehmanniae. Catalpol can elicit memory-improving effects through multiple mechanisms of action including antioxidant activity, anti-inflammatory activity, neurogenetic activity, and antiapoptotic activity [8]. (2) *β*-asarone, the major ingredient of Acorus tatarinowii Schott, could attenuate A*β*_1–40_-induced neuronal apoptosis in hippocampus by reversal downregulation of Bcl-2, Bcl-w, caspase-3 activation, and c-Jun N-terminal kinase (JNK) phosphorylation [30]. (3) Radix Ophiopogonis can promote learning and memory in haloperidol-induced aging rats and its mechanism is related to upregulating expression of antioxidase [31]. (4) The water extract of Poria cocos can enhance hippocampal long-term potentiation (LTP) and improve scopolamine-induced spatial memory impairment in rats [8]. (5) Matrine is one of the main active components that is extracted from the dry roots of* Sophora flavescens*. Matrine injection can decrease the level of IL-1B in the AD rats caused by the ibotenic acid and improve the pathological ultrastructural changes of the central nervous system [32]. (6) Paeonol, a constituent of the bark of the* Paeonia suffruticosa*, can increase levels of cortical cytochrome oxidase and vascular actin and improve learning and memory in a rat model of AD with injection of A*β*_1–42_ [33]. (7) The timosaponins, one group of the two major components of Anemarrhean asphodeloides Bge, can enhance the learning and memory capacities in rats with A*β*_25–35_-induced dementia, presumably in relation to their actions to promote the scavenging of the free radicals [34]. (8) Paeoniflorin, a monoterpene glycoside isolated from the aqueous extract of the dry root of Paeonia, possessed the neuroprotective effect of prevention of the neurotoxicity induced by A*β*_1–42_ the activity in a rat model of hippocampal dysfunction of AD [35]. (9) Dendrobium alkaloids, extracted from* Dendrobium nobile* Lindl., can improve lipopolysaccharide- (LPS-) induced memory impairment in rats, and this effect is related to prevent overexpression of tumor necrosis factor receptor 1 via inhibition of phosphorylated p38 mitogen-activated protein kinases and the downstream nuclear factor kappa-B signal pathway [36]. The alkaloids enriched extract from* Dendrobium nobile* Lindl. can attenuate LPS-induced hyperphosphorylation of tau protein in rat's hippocampus and protect against LPS-induced apoptosis in rat brain [37]. Therefore, from modern medicine's perspective, FMJ has significant effect on dementias and is potentially used to treat AD.

In conclusion, our findings demonstrated that FMJ exerts a significant improvement of learning and memory on rats insulted by A*β*_1–40_ and exhibits antiapoptotic effects, suggesting that FMJ could be used as a potential therapeutic formula for AD.

## References

[1] Rafii MS, Aisen PS (2009). Recent developments in Alzheimer's disease therapeutics. *BMC Medicine*.

[2] Alzheimer's Disease International World Alzheimer Report 2013. http://www.alz.co.uk/research/world-report-2013.

[3] Haas C (2012). Strategies, development, and pitfalls of therapeutic options for Alzheimer’s disease. *Journal of Alzheimer’s Disease*.

[4] Chan KY, Wang W, Wu JJ (2013). Epidemiology of alzheimer's disease and other forms of dementia in China, 1990–2010: a systematic review and analysis. *The Lancet*.

[5] Wang Y, Lin X, Zheng G (2011). Traditional Chinese medicine for Parkinson's disease in china and beyond. *Journal of Alternative and Complementary Medicine*.

[6] Jesky R, Hailong C (2011). Are herbal compounds the next frontier for alleviating learning and memory impairments? An integrative look at memory, dementia and the promising therapeutics of traditional Chinese medicines. *Phytotherapy Research*.

[7] Man SC, Durairajan SS, Kum WF (2008). Systematic review on the efficacy and safety of herbal medicines for Alzheimer’s disease. *Journal of Alzheimer’s Disease*.

[8] Lin Z, Gu J, Xiu J, Mi T, Dong J, Tiwari JK (2012). Traditional Chinese medicine for senile dementia. *Evidence-based Complementary and Alternative Medicine*.

[9] Zhang J-B, Jingyue Q (1991). * Complete Works of Jingyue*.

[10] Weiming Z, Haiyan H, Hong Z, Dandan M (2013). Study of the contents of serum Tau P-Tau and the clinical observation on effect of combination of western and traditional Chinese medicine on treating Alzheimer's disease. *Chinese Archives of Traditional Chinese Medicine*.

[11] Qianlin Y (1997). Experience introdution of Yan Dexi in treatment of senile dementia. *China Journal of Traditional Chinese Medicine and Pharmacy*.

[12] Wenruo C (2013). Traditional Chinese medicine for geriatric dementia. *Hubei Journal of Traditional Chinese Medicine*.

[13] Kang E-B, Kwon I-S, Koo J-H (2013). Treadmill exercise represses neuronal cell death and inflammation during A*β*-induced ER stress by regulating unfolded protein response in aged presenilin 2 mutant mice. *Apoptosis*.

[14] Popescu BO, Ankarcrona M (2004). Mechanisms of cell death in Alzheimer's disease: role of presenilins. *Journal of Alzheimer's Disease*.

[15] Paxinos G, Watson C (2005). *The Rat Brain in Stereotaxic Coordinates*.

[16] Morris R (1984). Developments of a water-maze procedure for studying spatial learning in the rat. *Journal of Neuroscience Methods*.

[17] Singh S, Kushwah AS, Singh R, Farswan M, Kaur R (2012). Current therapeutic strategy in Alzheimer's disease. *European Review for Medical and Pharmacological Sciences*.

[18] Neve RL, Robakis NK (1998). Alzheimer's disease: a re-examination of the amyloid hypothesis. *Trends in Neurosciences*.

[19] Walsh DM, Selkoe DJ (2004). Deciphering the molecular basis of memory failure in Alzheimer's disease. *Neuron*.

[20] Shin R, Ogino K, Kondo A (1997). Amyloid *β*-protein (A*β*) 1-40 but not A*β*1-42 contributes to the experimental formation of Alzheimer disease amyloid fibrils in rat brain. *Journal of Neuroscience*.

[21] Yamaguchi Y, Miyashita H, Tsunekawa H (2006). Effects of a novel cognitive enhancer, spiro[imidazo-[1,2-a]pyridine-3,2-indan]-2(3*H*)-one (ZSET1446), on learning impairments induced by amyloid-*β*
_1_-40 in the rat. *Journal of Pharmacology and Experimental Therapeutics*.

[22] Zou K, Kim D, Kakio A (2003). Amyloid *β*-protein (A*β*)1-40 protects neurons from damage induced by A*β*1-42 in culture and in rat brain. *Journal of Neurochemistry*.

[23] Hashimoto T, Adams KW, Fan Z, McLean PJ, Hyman BT (2011). Characterization of oligomer formation of amyloid-*β* peptide using a split-luciferase complementation assay. *The Journal of Biological Chemistry*.

[24] Zhang H, Zhang Y, Chen Y (2012). Appoptosin is a novel pro-apoptotic protein and mediates cell death in neurodegeneration. *Journal of Neuroscience*.

[25] Woo RS, Lee JH, Yu HN, Song DY, Baik TK (2010). Expression of ErbB4 in the apoptotic neurons of Alzheimer's disease brain. *Anatomy & Cell Biology*.

[26] Calissano P, Matrone C, Amadoro G (2009). Apoptosis and in vitro Alzheimer disease neuronal models. *Communicative and Integrative Biology*.

[27] D'Amelio M, Sheng M, Cecconi F (2012). Caspase-3 in the central nervous system: beyond apoptosis. *Trends in Neurosciences*.

[28] Kitamura Y, Shimohama S, Kamoshima W (1998). Alteration of proteins regulating apoptosis, Bcl-2, Bcl-x, Bax, Bak, Bad, ICH-1 and CPP32, in Alzheimer's disease. *Brain Research*.

[29] Drache B, Diehl GE, Beyreuther K, Perlmutter LS, König G (1997). Bcl-xl specific antibody labels activated microglia associated with Alzheimer's disease and other pathological states. *Journal of Neuroscience Research*.

[30] Geng Y, Li C, Liu J (2010). Beta-asarone improves cognitive function by suppressing neuronal apoptosis in the beta-amyloid hippocampus injection rats. *Biological and Pharmaceutical Bulletin*.

[31] Jieshu L, Liya H (2007). Effect of radix ophiopogonis and vitamin C on expression of anti-oxidase in Haloperidol—induced aging rats. *Tianjin Medical Journal*.

[32] Jingjing N, Zhongmin W, Shucai L, Zhu X (2006). Effect of Matrine injection on IL-1*β* level and ultrastructural changes of hippocampal neuron in Alzheimer's disease rat. *Chinese Journal of Neuroanatomy*.

[33] Zhou J, Zhou L, Hou D, Tang J, Sun J, Bondy SC (2011). Paeonol increases levels of cortical cytochrome oxidase and vascular actin and improves behavior in a rat model of Alzheimer's disease. *Brain Research*.

[34] Ouyang S, Sun LS, Guo SL, Liu X, Xu JP (2005). Effects of timosaponins on learning and memory abilities of rats with dementia induced by lateral cerebral ventricular injection of amyloid *β*- peptide. *Di Yi Jun Yi Da Xue Xue Bao*.

[35] Zhong S-Z, Ge Q-H, Li Q, Qu R, Ma S-P (2009). Peoniflorin attentuates *Aβ*
_(1−42)_-mediated neurotoxicity by regulating calcium homeostasis and ameliorating oxidative stress in hippocampus of rats. *Journal of the Neurological Sciences*.

[36] Li Y, Li F, Gong Q, Wu Q, Shi J (2011). Inhibitory effects of dendrobium alkaloids on memory impairment induced by lipopolysaccharide in rats. *Planta Medica*.

[37] Yang S, Gong Q, Wu Q, Li F, Lu Y, Shi J (2013). Alkaloids enriched extract from Dendrobium nobile Lindl. attenuates tau protein hyperphosphorylation and apoptosis induced by lipopolysaccharide in rat brain. *Phytomedicine*.

